# SSU rDNA Sequence Diversity and Seasonally Differentiated Distribution of Nanoplanktonic Ciliates in Neritic Bohai and Yellow Seas as Revealed by T-RFLP

**DOI:** 10.1371/journal.pone.0102640

**Published:** 2014-07-15

**Authors:** Jun Dong, Fei Shi, Han Li, Xiaoming Zhang, Xiaozhong Hu, Jun Gong

**Affiliations:** 1 Institute of Evolution and Marine Biodiversity, Ocean University of China, Qingdao, China; 2 Laboratory of Microbial Ecology and Matter Cycles, Yantai Institute of Coastal Zone Research, Chinese Academy of Sciences, Yantai, China; 3 School of Life Science, South China Normal University, Guangzhou, China; University of Connecticut, United States of America

## Abstract

Nanociliates have been frequently found to be important players in the marine microbial loop, however, little is known about their diversity and distribution in coastal ecosystems. We investigated the molecular diversity and distribution patterns of nanoplanktonic oligotrich and choreotrich (OC) ciliates in surface water of three neritic basins of northern China, the South Yellow Sea (SYS), North Yellow Sea (NYS), and Bohai Sea (BS) in June and November 2011. SSU rRNA gene clone libraries generated from three summertime samples (sites B38, B4 and H8) were analyzed and revealed a large novel ribotype diversity, of which many were low-abundant phylotypes belonging to the subclass Oligotrichia, but divergent from described morphospecies. Based on the data of terminal-restriction fragment length polymorphism (T-RFLP) analysis of all 35 samples, we found that the T-RF richness was generally higher in the SYS than in the BS, and negatively correlated with the molar ratio of P to Si. Overall, multidimensional scaling and permutational multivariate analysis of variance of the community turnover demonstrated a distinct seasonal pattern but no basin-to-basin differentiation across all samples. Nevertheless, significant community differences among basins were recognized in the winter dataset. Mantel tests showed that the environmental factors, P:Si ratio, water temperature and concentration of dissolved oxygen (DO), determined the community across all samples. However, both biogeographic distance and environment shaped the community in winter, with DO being the most important physicochemical factor. Our results indicate that the stoichiometric ratio of P:Si is a key factor, through which the phytoplankton community may be shaped, resulting in a cascade effect on the diversity and community composition of OC nanociliates in the N-rich, Si-limited coastal surface waters, and that the Yellow Sea Warm Current drives the nanociliate community, and possibly the microbial food webs, in the coastal ecosystem in winter.

## Introduction

Ciliates (phylum Ciliophora) are a morphologically diverse protozoan group with body lengths generally ranging from 10 to about 2,000 µm. Planktonic ciliates usually dominate the microzooplankton (20–200 µm) in different marine environments [1], [2], forming an important trophic link that channels carbon flow from the microbial to the classic food web [2]–[8].

As a component of planktonic ciliate assemblage, nano-sized ciliates (<20 µm) have also been recognized as significant grazers of bacterial, picophytoplankton and ultraphytoplankton production - a role once commonly attributed to the heterotrophic nanoflagellates [9]–[12]. It was observed that nanociliates comprised up to 57% of the total biomass of heterotrophic nanoplankton in diverse marine systems [10], 17% of oligotrich biomass in surface water of the northwest Mediterranean Sea [13], and about 36% or 23% of abundance in the oligotrophic Eastern Mediterranean [14], [15]. Recent studies showed that nano-sized oligotrich species contributed up to 88% of total ciliate abundance, and appeared to be the superior competitors in the oligotrophic Gulf of Aqaba, Red Sea, being able to utilize the dominant picoautotrophs efficiently [16]. In contrast, Dolan and Marrase found that nanociliates constituted only the 8% of the ciliate community in the Western Mediterranean [17]. In the Neva estuary of Baltic Sea, ciliate communities were composed essentially of pico- and nano-filterers (mostly algivorous) during the warm season, and became less important in the cold season; the most abundant size groups were small ciliates (20–30 µm) and nanociliates (<20 µm); nanociliates were represented essentially by different oligotrichids, scuticociliates, litostomatids and prostomes [18].

Despite the increasingly recognized ecological importance of nanociliates in different marine systems, their diversity, community composition and relationships with environmental conditions remain poorly understood. Further studies of this ecological group could be mainly hindered by the difficulties in morphological identification of species because of their small size and lack of distinct morphological characteristics. Nevertheless, culture-independent techniques can be used for studying nanociliate communities, as many microbial ecological studies have demonstrated. For instance, recent environmental rDNA surveys have revealed an unexpectedly large diversity and previously undescribed clades of small eukaryotes [19]–[28]. With the development of specific primers [29], [30], the diversity and distribution of choreotrichs and oligotrichs, the dominant groups of planktonic ciliates in the water column of coastal and open oceans, have been investigated using clone library, sequencing and denaturing gradient gel electrophoresis analysis of 18S rRNA [29], [31]–[33].

**Figure 1:**
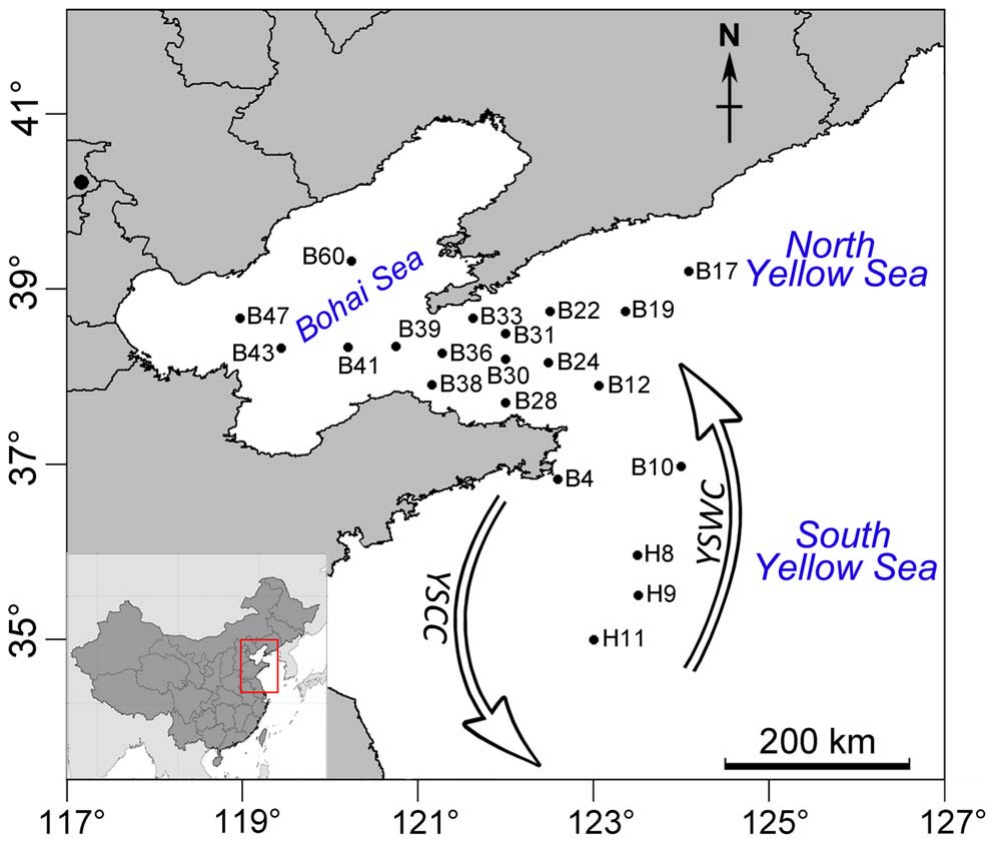
Illustration of the sampling sites. A map showing the sampling stations, the Yellow Sea Coastal Current (YSCC), and Warm Current (YSWC) in the South Yellow Sea (B4, 10; H8, 9 and 11), North Yellow Sea (B12, 17, 19, 22, 24, 28, 30, 31, 33 and 38) and Bohai Sea (B39, 41, 43, 47 and 60).

The highly productive Yellow Sea comprising the South (SYS) and North Yellow Sea (NYS) basins is one of the largest shallow continental shelf areas in the world, with an average depth of 44 m and maximum depth about 100 m. The Bohai Sea (BS) is a shallow embayment of the Yellow Sea with an average depth of 10 m. These three regions are physically and biologically linked. The Yellow Sea Warm Current (YSWC), a branch of the Kuroshio Current, and the Yellow Sea Coastal Current, play an important role in the water exchange in these regions [34]. The YSWC comes from the East China Sea with relatively high-temperature (>12°C) and high salinity (>33 psu) water flowing northward along the 124°E meridian and then westward into the Bohai Sea in the winter (Fig. 1). However, the molecular diversity and distribution of planktonic ciliates, including the nanociliates, have rarely been studied in these areas [35], [36].

In this study, we aimed to reveal the molecular diversity, spatiotemporal distribution pattern of nano-sized oligotrich and choreotrich (OC) ciliates in surface waters of the neritic oceans in two seasons using rDNA clone library construction, sequencing, and newly developed terminal restriction fragment length polymorphism (T-RFLP). We hypothesized that there were distinctly seasonal and spatial patterns of diversity and community composition of nanociliates, and their community structure was significantly influenced by the YSWC in winter.

## Methods

### Study area and sample collection

No specific permits were required for the described field studies. Summer (from 14 to 26 June 2011) and winter (from 21 November to 4 December 2011) cruises were carried out with R/V Dong Fang Hong 2 on the Bohai and Yellow Seas. From the initial grid of 110 stations, 17 were sampled in summer and 18 in winter. Only surface samples were analyzed here. A total of 21 stations were sampled, with 5 stations in the Bohai Sea, 11 in the North and 5 in the South Yellow Seas (34°59′–39°20′N, 118°58′ –124°06′ E, see Fig. 1). The field studies did not involve endangered or protected species. Surface water (∼2.5 L) was collected during the cruises using a Seabird 911 Plus CTD rosette (USA) fitted with 10 L Niskin bottles, prefiltered over a 200-µm mesh, followed by a 20-µm mesh to remove most of the mesozooplankton, microplankton and particles. Subsequently, the biomass of 2 L water samples was filtered by vacuum pressure onto a 0.2-µm-pore-sized membrane (PALL, Michigan, USA), which was then immediately put into a 2-ml cryotube and stored in liquid nitrogen. Water temperature (Temp), salinity (Sal), and dissolved oxygen concentration (DO) were obtained from the CTD sampler. Chlorophyll *a* (Chl-*a*) and pH was determined with an electronic probe (M5S, Hach, USA). Nutrients such as ammonium nitrogen (NH_4_–N), nitrate nitrogen (NO_3_–N), nitrite nitrogen (NO_2_–N), total nitrogen (TN), silicate (SiO_2_), and soluble reactive phosphorus (PO_4_–P), were determined with an automatic analyzer III (Seal, Germany).

### DNA extraction, PCR and T-RFLP analysis

DNA was extracted from filters cut into small pieces using the Fast DNA Spin Kit for soil (MP Biomedicals, Irvine, CA, USA) following the standard protocol. For T-RFLP analysis, SSU rRNA genes of nanoplanktonic ciliates were amplified with a nested PCR approach. Eukaryotic SSU sequences were amplified with primer Euk528F: 5′-GCG GTA ATT CCA GCT CCA A-3′ and U1492R: 5′-GGT TAC CTT GTT ACG ACT T-3′ [37]. The 25-µl PCR reaction solution for each sample contained 1.0 µl of each primer (10 µM), 2.5 µl of 10× PCR Buffer, 2.5 µl of 25 mM MgCl_2_, 0.5 µl 10 nM dNTP mix, 0.2 µl of 1 U *Taq* DNA polymerase (Fermentas, Thermo Scientific), 16.3 µl RNase-free water and 1 µl of template DNA. The first round PCR reaction consisted of an initial step of 3 min at 94°C, followed by 35 cycles of denaturation at 95°C for 45 s, annealing at 55°C for 1 min and extension at 72°C for 2 min, and finally 10 min of extension at 72°C. The second round PCR consisted of OC ciliate-specific primer 1199+ with 6-carboxyfluorescein (6-FAM) labeled: 5′-GCC GAC TCG GGA TCG GGG GC-3′ and 1765-: 5′-CCC CAK CAC GAC DCM TAT TGC TG-3′ [29], which targeted a partial fragment of the first-round PCR product. Cycling conditions for OC-specific primers were as follows: an initial denaturation at 98°C for 2 min, 35 cycles of 98°C for 15 s, 55°C for 30 s and 72°C for 1.5 min, then a 10 min extension at 72°C [29]. Three separate 25 µl reactions were run and the products were pooled to minimize PCR bias. This PCR procedure is expected to amplify a 566-bp region.

PCR products were checked for quality by electrophoresis in 1% agarose gel, and purified with TIAN Quick Midi Purification Kit (Tiangen, Beijing, China). The DNA yield was quantified with a NanoDrop 2000C Spectrophotometer (Thermo, Wilmington, DE, USA). The 15 µl digestion solution, which contained 7.5 µl purified PCR product (each with about 20 ng DNA) and 7.5 µl digestion buffers with two restriction enzymes *BfaI* and *RsaI* (Fermentas, Thermo Scientific), was incubated at 37°C for 1 hour according to the manufacturer’s instruction. These two enzymes were selected using the online tool MiCA (Microbial Community Analysis) [38] based on the *in silico* performance of a range of enzymes in digesting known SSU sequences of OC ciliates.

The labeled terminal restriction fragments (T-RFs) were analyzed using a 3130×L genetic analyzer (Applied Biosystems). This results in the generation of profiles in which the number of peaks indicates the number of different T-RFs present, while the heights and areas of peaks indicate relative abundances. The baseline threshold for signal detection was set to 50 fluorescence intensity units to eliminate background interference. Only peaks representing T-RFs with lengths between 50 and 560 bp were considered for the following analysis. Minor peaks with a relative abundance <1% of the total were excluded and the remaining peaks were presumed to represent phylotypes of the nano-sized OC ciliates.

### Statistical analyses

Data of relative abundance generated from T-RFLP were log-transformed to calculate a Bray–Curtis similarity matrix for all the 35 samples. Multidimensional scaling (MDS) plots were made using the software PRIMER 6/PERMANOVA+ (PRIMER-E, UK). Variability in community structure among samples in terms of grouping factors (e.g. season, location) was statistically tested by Permutational Multivariate Analysis of Variance (PERMANOVA) [39]. Both weighted (with relative abundance of T-RFs) and unweighted (with the presence and absence of T-RFs) similarity matrices were subjected to PERMANOVA. Spearman’s rank correlations between T-RF number and variables were performed with software SPSS (SPSS Inc., USA). Simple and partial Mantel tests were performed to determine the correlation coefficients between Bray–Curtis community dissimilarity and geographic and environmental distances (Temp, Sal, pH, DO, PO_4_-P, SiO_2_, NH_4_-N, NO_3_-N, NO_2_-N, molar ratios of P:Si and N:P). *P*-values were obtained by 999 permutations.

### Cloning, sequencing and phylogenetic analyses

By visually inspecting their distances in the MDS plot as indications of differences in community structure, we selected three representative samples H8S, B38S and B4S for clone library construction. PCR, product purification were performed as described above. Cloning and Sanger sequencing were conducted as previously described [40]. One hundred clones were randomly selected and sequenced for each library. The obtained sequences (about 566-bp in length) were aligned using the program MAFFT with default parameters [41]. Three programs Bellerophon [42], KeyDNATools [43] and BLAST against GenBank [44] were separately used to detect possible chimeric sequences, resulting in a combined set of 24 (8%) identified chimeras, which were then removed from subsequent analysis. Sequences have been deposited in Genbank under accession numbers KF613718– KF613993. The theoretical *in silico* enzyme digestion analysis was performed with the sequence dataset obtained from the three clone libraries using MiCA.

To assign operational taxonomic units (OTUs), sequences were aligned using MAFFT and distance matrices based on Jukes-Cantor model were created with the DNAdist program in Phylip 3.66 [45]. A cutoff of 98% sequence similarity and the furthest neighbor algorithm were applied to collapse similar sequences into OTUs. The rarefaction curves and OTU richness were calculated using DOTUR [46].

A total of 45 SSU rRNA gene sequences of morphologically identified OC ciliate species retrieved from GenBank were aligned with the 59 representative sequences of nanociliate OTUs using MAFFT. The alignment with 510 bp sites was used for building phylogenetic trees. PhyML 3.0 [47] was used to build a maximum likelihood (ML) tree with the best model GTR+I (0.5191)+G (0.4676) selected by MrModeltest 2 [48], and 1000 non-parametric bootstrap was applied. A neighbor-joining (NJ) tree was constructed based on Kimura 2-parameter model using MEGA 5.05 [49], with 1000 bootstrap replicates.

## Results

### Physicochemical properties

Seventeen and 18 stations were surveyed during the summer and winter cruises, respectively. Location, depths and physicochemical properties of water samples are given in Table S1. The average water temperature in summer (17.3±2.4°C, mean±SD, n = 17) was significantly higher than that in winter (13.1±1.1°C, n = 18) (t-test, *P*<0.001). For the three regions, temperature was significantly higher in the SYS (14.5±0.4°C) than the NYS (12.6±0.6°C) or BS (12.5±0.7°C) in the winter (t-test, *P*<0.01), but no statistical differences were found among regions in the summer (16.6–18.1°C). The salinity of all sampling stations was not significantly different between summer (31.4 psu) and winter cruises (31.2 psu). However, the SYS samples had a higher salinity (32.0±0.27 psu) than NYS (31.2±0.25 psu) and BS (31.1±0.12 psu) in summer (t-test, *P*<0.001). The Chl-*a* concentration in the summer (3.1±1.3 µg/l) was significantly higher than that in the winter (1.2±0.6 µg/l) (t-test, *P*<0.001) for the whole regions, and among regions in the summer (NYS, 1.6±0.9 µg/l; BS, 4.5±0.0 µg/l; SYS, 3.4±0.8 µg/l), but not significantly different among regions in the winter (BS, 0.6±0.2 µg/l; NYS, 1.5±0.6 µg/l; SYS, 1.4±0.2 µg/l) (ANOVA, *P*>0.05). As for nutrients, the ratios of N:P were always higher than the Redfield value (16∶1), with relatively lower levels of SiO_2_ in summer (0.13–1.23 µmol/l) than in winter (0.38–4.17 µmol/l). The ratios of P:Si were generally higher than the Redfield value (1∶15) in summer but lower in winter, suggesting a seasonal shift from Si-limitation to P-limitation to phytoplankton in surface waters.

### Clone libraries

**Figure 2:**
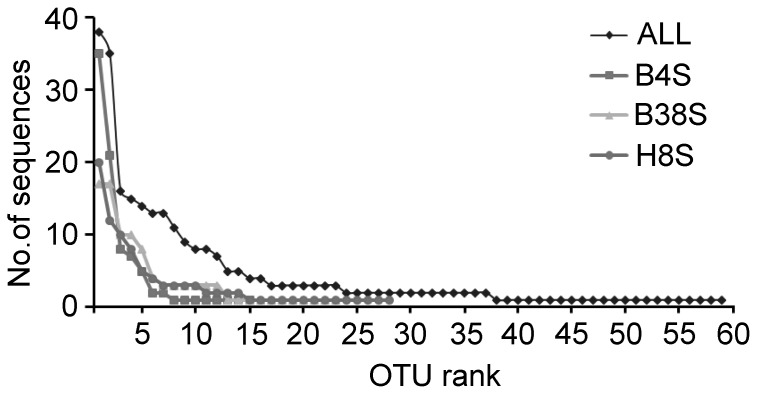
Abundance rank of OTUs. Rank abundance curves of operational taxonomic units (OTUs) and sequences derived from the three clone libraries of the summertime samples collected from stations H8, B4, and B38. OTUs were defined at a cutoff of 98% sequence identity.

A total of 276 SSU rRNA gene sequences and 59 OTUs were obtained from the three clone libraries for the samples B38S, B4S and H8S, which were collected in the summer of 2011 (Table S2). The rank abundance curves showed there were many rare OTUs and fewer abundant ones across the three samples (Fig. 2). If 1% relative abundance cutoff (e.g. <3 sequences) was applied for rare OTUs, then 23 (about 39%) abundant OTUs accounted for 82% sequence abundance, whereas 36 (61%) rare OTUs accounted only 18% sequence abundance. There were 22 OTUs represented by a single sequence in the three libraries.

**Figure 3:**
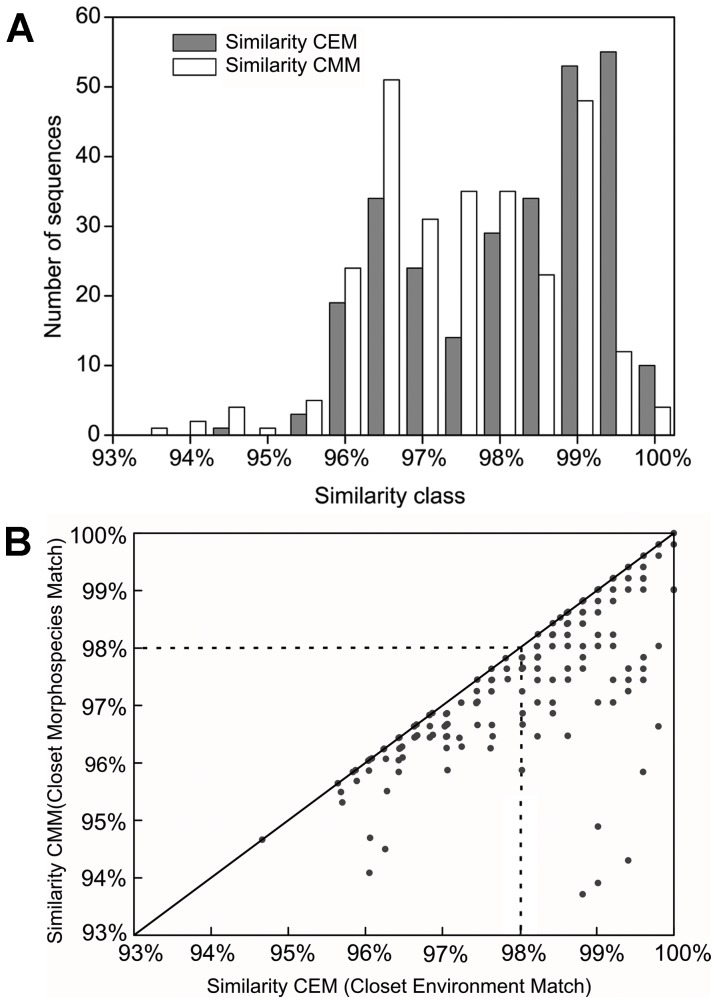
Histogram and dispersion plots of the CEM and CMM similarities for sequences. Novelty analysis of the 276 sequences of oligotrich and choreotrich 18S rRNA genes amplified from environmental DNA of the nanoplanktonic fractions. (A) Histogram showing the distribution of similarities against closest environmental match (CEM) and closest morphospecies match (CMM) of all sequences, in 0.5% similarity classes. (B) Dispersion plot of the CEM and CMM similarities for each sequence.

BLAST against GenBank using obtained sequences was performed, and the closest matches of both taxonomically classified (closest morphospecies match, CMM) and environmental sequences (closest environmental match, CEM) were recorded (Fig. 3; Table S2). The similarity distribution against CEM peaked at 99.5 and 99%, whereas the similarity distribution against CMM was highest at 96.5% and 99% (Fig. 3A). The similarity of CMM had never been higher than that of CEM (Fig. 3B). The closest matches of taxonomically classified taxa all belonged to oligotrich and choreotrichs, with similarities ranging from about 93.5% to 100% (Table S2; Fig. 3A). Most OTUs (81.4%) appeared to be affiliated with Oligotrichia. Forty-four OTUs (74.6%) exhibited a sequence similarity lower than 98%, and there were 10 OTUs showing lower (<96%) similarities with described species. The most frequently detected OTU in the three libraries (OTU1) had a similarity of 99.6% to the species *Strombidium biarmatum* (accession No. AY541684 [50]).

**Figure 4:**
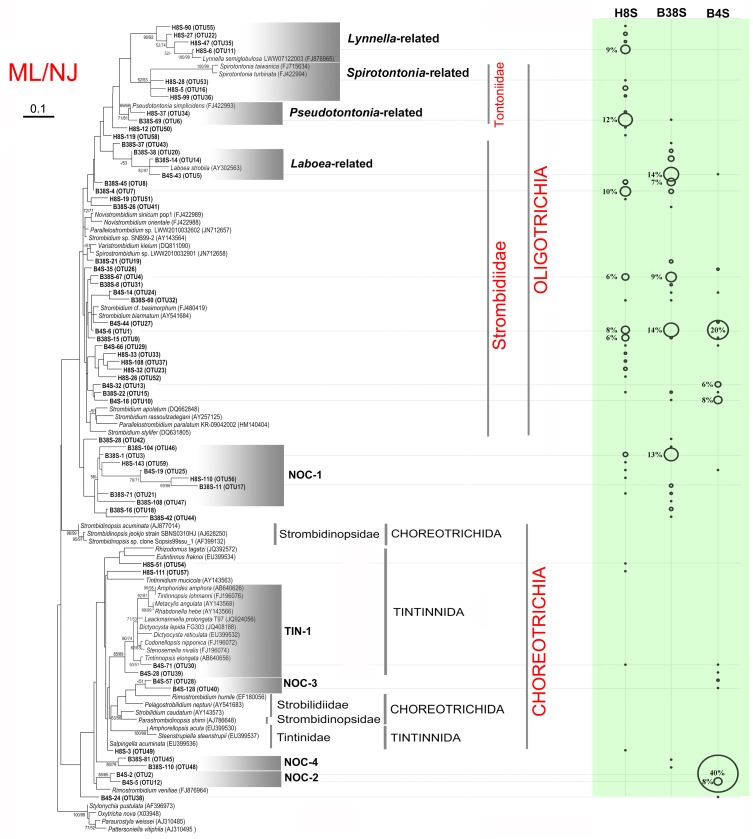
A consensus of maximum likelihood (ML) and neighbor-joining (NJ) phylogenetic trees based on 18S rRNA gene sequences. Showing the phylogenetic placements of representatives of operational taxonomic units and described species of oligotrich and choreotrichs. Sequences newly obtained in this study are in bold. Relative abundances of OTUs are indicated by circles of proprotional size on the right. Only bootstrap values no less than 50% are shown. Accession numbers are provided in brackets. The scale bar indicates ten nucleotide changes in 100 sites.

At the 98% sequence similarity cutoff, there were 28, 23 and 15 OTUs in H8S, B38S and B4S libraries, respectively (Fig. S1). Only three phylotypes (OTU1, 9 and 15) were shared in the three clone libraries. OTU1 and OTU5 were the most and equally abundant (14%) in the sample B38S, whereas OTU2 (40%) and OTU6 (12%) dominated in the samples B4S and H8S, respectively (Fig. 4).

### Phylogenetic analysis

Phylogenetic analyses were performed to infer the systematic placements of the representative sequences of these OTUs. The ML and NJ trees show similar topologies (Fig. 4). Overall, about 73% (43 out of 59) OTUs obtained in this study were placed closer to the subclass Oligotrichia than to the subclass Choreotrichia in the phylogenetic trees (Fig. 4). Nevertheless, there were only 8 OTUs clustering and forming moderately to highly supported monophyletic clades with described oligotrich species. These included 3 OTUs with *Spirotontonia* (ML 52%, NJ 53%), 2 OTUs with *Pseudotontonia* (ML 71%, NJ 81%), and 3 OTUs with *Laboea* (ML <50%, NJ 53%) (Fig. 4). In addition to the 3 *Laboea*-related OTUs, there were 21 OTUs grouping with typical taxa of the family *Strombidiidae* (e.g. *Strombidium*, *Spirostrombidium* and *Varistrombidium*) (Fig. 4; Table S2). However, the bootstrap supports for these topologies were lower than 50%, and their sequence identities varied greatly from 96.3% to 99.6% (Fig. 4).

Interestingly, a major clade, which comprised 11 newly obtained OTUs, was identified as a basal lineage to strombidiids and tontoniids. Within this clade, a monophyletic subgroup comprising 8 OTUs (i.e. OTU3, 17, 21, 25, 46, 47, 56 and 59), designated as Nanoplanktonic Ciliate clade 1 (NOC-1), was moderately supported in the ML tree (58%). However, the phylogenetic relationship between NOC-1 and other oligotrichs was not resolved due to the low (<50%) bootstrap supports.

Four OTUs grouped with a choreotrichid species *Lynnella semiglobulosa* with high ML (90%) and NJ (92%) bootstrap supports. Although this *Lynnella*-related clade branched off from tontoniids, their phylogenetic relationship was not supported. The remaining 12 OTUs mostly clustered with tintinnids and choreotrichids, sharing identities ranging from 94.7% to 99.2% with closest matches (Table S2). Among these, 2 OTUs were placed within a well-supported clade of *Tintinnida* (TIN-1, see Fig. 4). Another three clades, NOC-2, -3 and -4, each comprising two OTUs, were moderately to highly supported in either the ML or NJ trees. However, their relationships with other described choreotrichs were not supported statistically.

### Seasonal and spatial patterns of diversity and community structure revealed using T-RFLP

T-RFLP profiles targeting the 18S rRNA genes of nanoplanktonic oligotrichs and choreotrichs were determined for all the 35 samples. Based on the clone sequences from the three summertime samples, the *in silico* digestion analysis showed that these two enzymes would cut all these sequences. However, the corresponding T-RFLP profiles did not match accurately with the libraries. For example, the most presented OUT1 (corresponding to T-RF 311 bp) was present in all the libraries, but absent from the profiles for two of the three samples (B4S and B38S). On the other hand, some distinct T-RFs (e.g., 53, 55, 78, 93, 191, 192, 506 bp) with a relative proportion ranging 3∼39% were observed in the profiles of the three samples, but could not be matched with a sequence from the three libraries. For all the 35 samples, there were 30 T-RFs detected more than once, among which 18 T-RFs were exclusively found in either the summertime (e.g., 78, 93, 164, 185, 191, 304, 319, and 322 bp) or the wintertime (e.g., 73, 128, 198, 357, 368, 380 and 509 bp). Again, a few of these T-RFs could be linked to sequences in the three libraries and GenBank.

**Figure 5:**
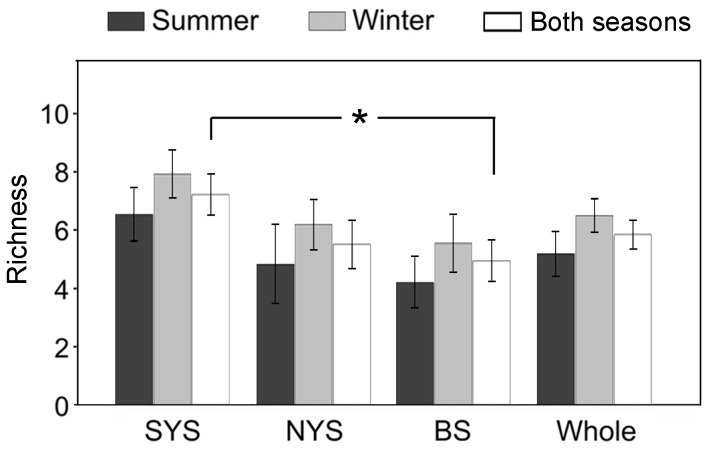
Spatial and seasonal variations of terminal restricted fragment numbers. Data were derived from water samples from South Yellow Sea (SYS), North Yellow Sea (NYS), and Bohai Sea (BS) and the combined area (Whole). The asterisk indicates significant difference (*P*<0.05).

The T-RFLP pattern revealed in total 72 T-RFs, ranging from 1 (in the sample B22S) to 14 (in the sample B38S) T-RFs per sample. The most frequently occurring T-RF was 311 bp long and was found in 28 out of the 35 samples. There were 41 unique T-RFs, which appeared only once in all the samples. In each basin, the T-RF richness appeared higher in winter than in summer, and decreased from SYS to NYS and BS (Fig. 5). The richness was significantly higher in SYS than in BS across two seasons (*P*<0.10).

**Figure 6:**
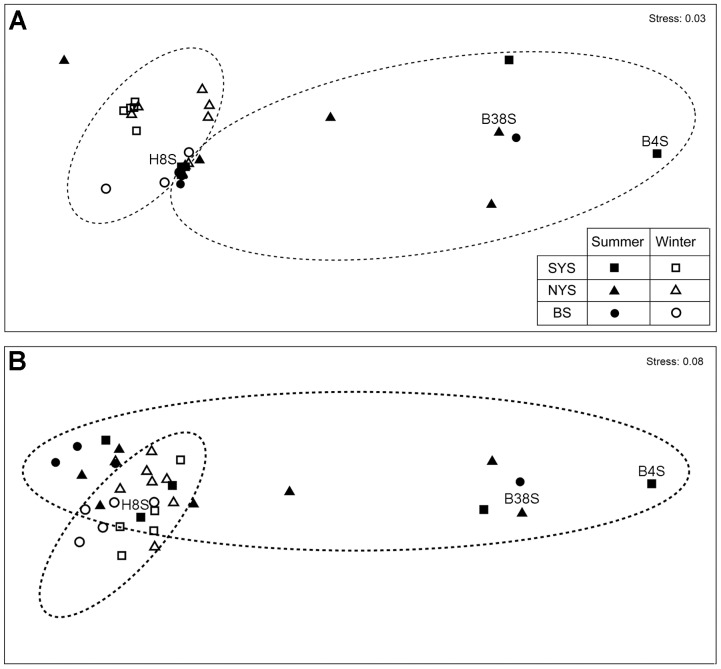
Plots of multi-dimensional scaling ordination based on weighted (A) and unweighted (B) community distance matrices inferred from terminal-restriction fragment length polymorphism analysis of 35 surface water samples. A. Circles of dash lines indicate that samples are distinctly clustered into the summer and winter groups. Three representative samples (H8S, B38S and B4S) selected for clone library analysis were labelled.

The community structure of nanociliates showed a distinct seasonality according to the MDS ordination (Fig. 6), which was supported by PERMANOVA, either based on weighted (*P* = 0.002) or unweighted (*P* = 0.001) community distance matrices (Table 1). However, significant differences among basins were not supported (*P*>0.10). To further test that there were spatial patterns, the summer and winter samples were then analyzed separately (Table 1). The summertime samples were loosely spaced in the plots, and samples of two distant basins (e.g., BS and NYS) were much more closely located than two neighboring basins (e.g. SYS and NYS) (Fig. 6), indicating a strong spatial heterogeneity but lack of basin-to-basin spatial patterns. This was consistent with the PERMANOVA results, which rejected the hypothesis that these summertime samples were significantly different among basins (*P*>0.86). In contrast, the community composition of nanociliates in winter was significantly different among basins (weighted, *P* = 0.001; unweighted, *P* = 0.002; Table 1), showing a distinct spatial pattern of distribution across the three basins investigated.

**Table 1:** PERMANOVA results of nanociliate community structure in different regions and seasons.

Groups	Weighted		Unweighted	
	Pseudo-F	*P*	Pseudo-F	*P*
Season (all samples)	1.949	**0.002**	2.056	**0.001**
Basin (all samples)	0.454	0.136	1.181	0.268
Basin (summer only)	0.486	0.927	0.638	0.861
Basin (winter only)	5.165	**0.001**	2.711	**0.002**
SYS vs. NYS	2.349	**0.011**	1.635	**0.012**
SYS vs. BS	3.209	**0.011**	1.571	**0.043**
NYS vs. BS	1.619	**0.032**	1.709	**0.017**

Community similarities are based on relative abundance of T-RFs (weighted), and the presence or absence (unweighted) of T-RFs.

### Correlations between T-RF richness, community turnover, and environmental factors

Significant correlations were only detected between the molar ratio of P:Si and the richness (Spearman’s ρ = −0.366, *P*<0.05) To disentangle the relative influence of environmental factors and geographic distance on the distribution of nanociliate communities, both simple and partial Mantel tests were performed (Table 2). For the dataset of all samples, no geography was observed, but P:Si, Temp and DO showed significant effects on community turnover, regardless of weighted or unweighted Bray-Curtis distance matrices used (*P*<0.05), and their effects remained when geographic distance was constrained. For the winter samples, significant correlations were found between community variations and geographic distance (simple Mantel, *r* = 4.448, *P*<0.001 and *r* = 0.254, *P*<0.01, for weighted and unweighted matrices, respectively), and several environmental factors (*P*<0.05). However, when geographic distance was controlled, only DO (partial Mantel, *r* = 0.249, *P*<0.01 and *r* = 0.209, *P*<0.05 for weighted and unweighted matrices, respectively) was significantly correlated with community changes (Table 2).

**Table 2:** Simple and partial Mantel tests showing the top ranked correlation coefficients (*r*) between geographic distance and environmental factors and (weighted or unweighted) community dissimilarity matrices derived from T-RFLP.

		Weighted		Unweighted	
Dataset	Variable	Simple (*r*)	Partial (*r*)	Simple (*r*)	Partial (*r*)
All samples	P:Si	0.262^*^	0.262^*^	0.270^*^	0.270^*^
	Temp	0.193^*^	0.194^*^	0.222^**^	0.223^**^
	DO	0.105^*^	0.105^*^	0.225^***^	0.225^***^
	Chl-*a*	0.098	0.099	0.270^*^	0.270^*^
	TN	−0.053	−0.054	0.248^**^	0.249^**^
	Distance	−0.011		−0.008	
Summer samples	P:Si	0.192	0.209	0.063	0.078
	pH	0.140	0.136	0.108	0.105
	TN	0.126	0.112	0.025	0.009
	SiO_2_	0.077	0.101	0.033	0.056
	Distance	−0.172		−0.174	
Winter samples	Distance	0.448^***^		0.254^**^	
	Temp	0.400^**^	0.242^**^	0.103	−0.018
	DO	0.357^**^	0.249^**^	0.274^*^	0.209^*^
	Chl-*a*	0.239^*^	0.138	0.155	0.093
	Sal	0.208^*^	−0.070	0.131	−0.020
	pH	0.095	−0.064	0.105	0.156

Partial Mantel tests were performed by controlling for geographic distance.

Significant correlations (*P*<0.05) are highlighted in bold. *, *P*<0.05; **, *P*<0.01; ***, *P*<0.001. Abbreviations: Temp, temperature; DO, dissolved oxygen; SiO_2_, silicate; NH_4_-N, ammonium nitrogen; TN, Total Nitrogen; Sal, salinity; Chl-*a*, Chlorophyll *a*.

## Discussion

Ecological studies of marine ciliate assemblages have traditionally relied on morphology-based methodologies [51]. In a large-scale ecological survey of ciliates, in particular the nano-sized assemblage, there are difficulties in working with these organisms. First of all, accurate identification of ciliates can be laborious, since both light microscopical observation and protargol silver-staining should be preferably carried out to reveal living features (e.g. size, shape, features of lorica, and behavior etc.) and infraciliature (the pattern of ciliation). Secondly, because of their small size, different species of nanociliates may exhibit similar living features under light microscopical observations, which may result in unreliable isolation and subsequently characterization of the populations. Thirdly, oligotrich species are fragile, and can be easily broken on slides during living observation and fixation for protargol silver staining. In this regard, the rDNA-based clone library and profiling techniques have provided efficient tools for large-scale survey of the diversity and composition of small ciliate assemblages.

### Large contribution of novel and low-abundant ribotypes within small oligotrich and choreotrich ciliates

In this study, we found a large number of low-abundant (relative abundance <1%) and only a few dominant OTUs across the three clone libraries for nano-sized OC ciliates (Fig. 4; Table S2). This is not unexpected as similar results were obtained in previous studies of the same target taxa in different planktonic fractions (>20 or >3 µm) [29], [31]–[33]. One possibility is that these low-abundant OTUs may be artifacts due to the intragenomic variation of rDNA, as our recent study has demonstrated that there are numerous rare rDNA haplotypes in a single cell of ciliates [40]. This finding may explain previous observations that the richness of OTUs (frequently defined with a 1% sequence cutoff) is consistently higher than the morphology-based assessments [29], [31]–[33], [52]. However, the 2% sequence cutoff we used in detected in this study should, to some extent, offset the intragenomic variation and thus reflect population or species level diversity more accurately. Consequently, the existence of these novel clades comprising low-abundant OTUs indicates that there is a hidden ribotype diversity of oligotrichs and choreotrichs waiting to be described. In addition, some OTUs (e.g. OTU5, OTU10) were rare in one sample but abundant in another, suggesting these low-abundant taxa may play an increasing role in ecological functioning under certain conditions [53].

The OTUs affiliated with the subclass Oligotrichia were consistently richer and more abundant than these clustered with the subclass Choreotrichia in the three clone libraries (Fig. 4; Table S2). This may reflect the nano-sized nature of the planktonic samples we investigated, because the choreotrich ciliates are loricate and usually conspicuously large and hence most their cells are less likely to be concentrated and collected through the 20-µm-sized mesh filtration procedure. In fact, many abundant OTUs detected in this study are closely related to small-sized oligotrich morphospecies. For example, two OTUs form a monophyletic clade, and show over 98% sequence similarities with *Pseudotontonia simplicidens*, whose body size is approximately 25–50×15–30 µm *in* *vivo* [54]. The newly-born or smaller-sized individuals of *Strombidium basimorphum* (30–60×35–60 µm), *Lynnella semiglobulosa* (40–50×65–75 µm) [55], and *Laboea strobila* (50–150×25–75 µm), morphospecies phylogenetically related to many nanociliate OTUs, can be also included in the nanoplanktonic fraction and detected. Nevertheless, the bias of the primer set (1199+/1765–) may also contribute to the over-representation of the oligotrichs [29], [31]–[33].

As OC ciliates may possess extremely high copy numbers of rDNA in a single cell [40], DNA contained within small amounts of nuclear debris from larger species or preserved DNA are likely detected via PCR amplification, and thus may contribute to the diversity of nanociliates estimated in this study. Nevertheless, we assume that this part of diversity could be minor, as demonstrated by the presence but rarity of tintiniid sequences in two of the three libraries (H8S and B4S). Furthermore, for large-sized morphospecies of oligotrichs and choreotrichs, there has an increasing large number of 18S rDNA being sequenced and deposited in GenBank recently, but still, a majority of nanociliate OTUs (e.g. OTU1, 4 and 7, OTU3 of NOC-1, and OTU2 and 12 of NOC-2) obtained in this study showed loose phylogenetic relationships with publicly available sequences (and also our sequences from single-cell PCR of individual “large” morphospecies obtained during the same cruises, unpublished data), supporting that the majority diversity recovered in this study does come from the nano-sized fraction. Nevertheless, the morphological identity of these novel ribotypes of nanociliates remains to be revealed by either classical morphological taxonomy or by linking sequence information to morphotypes using molecular approaches (e.g., fluorescence *in situ* hybridization).

### Lower diversity in Bohai Sea than in South Yellow Sea

Based on the T-RFLP profiles of all samples, we found spatial rather than seasonal variations in number of nanociliate phylotypes in the surface waters of the three basins, with generally lower phylotype richness in BS than in SYS (Fig. 5). The lower diversity may be indicative of high levels of nutrients and pollutions in BS than other two basins. More specifically, correlation analysis demonstrated a negative relationship between diversity of nanociliates and P:Si ratio, suggesting that the diversity of nanociliates may be related to diatoms, since the growth of diatom is affected by N:P:Si ratios [56]. The Si concentrations in most of our samples were lower than 2 µM (Table S1), which would lead to dominance of flagellates over diatoms in the phytoplankton community [57]. According to the Redfield-Brzezinski nutrient ratio C:Si:N:P = 106∶15∶16∶1 [58], the Si was the most limited element for diatoms in most samples we studied. In this case, nanociliates may be relying on bacteria and picoeukaryotes for food. Thus, the diversity of nanociliates, and perhaps the microbial food webs, appeared to be controlled by bottom-up processes in which the growth of diatoms represents the limiting factor in N-rich and Si-limited coastal waters.

### Distinct variations in community composition and major drivers

The results of PERMANOVA lend support for a distinct seasonal pattern for all samples and a spatial pattern in winter (Table 1). These differential patterns of phylotype richness and community composition can be explained by the higher sensitivity of community-based multivariate analyses than univariate ones (diversity metrics) in detecting changes in communities, as communities with different species composition may exhibit similar diversity indices [59], [60]. It has been postulated that open-water marine systems may lack the large seasonal change in tintinnid assemblages known from coastal systems [61]. However, our results agree with the morphology-based study by Mironova et al. [18] in that the seasonal changes of nanociliate community structure was more significant than the spatial variation in the open-water system. It is possible that the nanociliates in open waters follow a seasonal encystment-excystment cycle as previously observed for *Strombidium conicum* [62].

Our results also shed light on biogeography of marine protists. Based on either weighted or unweighted community distance matrices, the Mantel tests indicated that the distribution of nano-sized OC ciliate assemblages in surface water in the studied area was largely governed by environment rather than biogeographic distance. The ratio of P:Si may be an indicator of types of nutrient limitation, which governs phytoplankton community, and impacts the nano-sized OC ciliate assemblages through the microbial trophic links. In addition, temperature and DO are also important environmental factors. Nevertheless, in the winter, when the water samples had a higher concentration of silicate and lower P:Si ratios than in the summer, the nanociliate assemblages exhibited a distinct distance-decay, and DO was the key environmental factor in shaping the spatial distribution of nanociliates in the three basins (Table 2). In contrast, no spatial pattern was observed in summer. This situation matches well with the occurrence of the YSWC, which is only detectable in winter through carrying warm waters that mix with cooler local waters during its flow from the SYS to the NYS and BS. Therefore, these results support our hypothesis that the community composition of nanociliates is influenced by the YSWC in winter.

### Limitations

There are potential caveats for using rDNA-based methodologies in characterizing changes in ciliate communities. Since rDNA copy number may be highly different among ciliate species [40], and nested PCR with many cycles may lead to biases in this study, the community structure based on relative abundance of rDNA sequences may not reflect accurately the biological community in nature. Nevertheless, our hypothesis testing based on both weighted and unweighted community distance matrices yielded generally consistent results, indicating that the copy number problem should not affect the conclusion drawn in this study.

Not all T-RFs in the profiles matched with *in silico* results of clone sequences, as we shown for the three summertime samples in this study, reflecting a caveat for using T-RFLP analysis in community ecology of ciliates. A single ciliate species may have slightly different SSU rDNA haplotypes with extensive single nucleotide polymorphisms [40], of which some may possess the same restrict enzyme sites and collectively form a T-RF. This chance increases when many species within a community are analyzed. However, these T-RFs may not be identified *in silico* due to low coverage of the clone library. This suggests that T-RF-based taxon richness should be interpreted with caution. The links between these genetic drift of ciliate community rDNA revealed by T-RFLP and environments remain largely unknown. However, a recent study has shown that changes of dominant taxa in protist communities can be captured with T-RFLP, which, as compared with sequencing, is relatively rapid, inexpensive, and still useful for analyzing large ecological data [63].

## Concluding Remarks

In conclusion, we studied for the first time the molecular diversity, spatial and seasonal distribution and environmental drivers of the nano-sized fraction of oligotrich and choreotrich ciliates in an N-rich and Si-limited neritic ecosystem. Our study shows that in surface waters there are previously unrecognized new clades of oligotrich and choreotrichs. With the newly developed T-RFLP protocol, we found a significantly higher diversity of nanociliate phylotypes in South Yellow Sea than in Bohai Sea, two basins with contrasting ratios of P:Si. Overall, their community composition is shaped by environmental parameters rather than geographic distance, with a distinct seasonal pattern in the Bohai, North and South Yellow Seas. However, in winter, the effects of both geographic distance and concentration of dissolved oxygen on community turnover are distinct, supporting the idea that the Yellow Sea Warm Current carrying warmer and less oxygenated waters plays an important role in structuring the nanoplanktonic assemblages, and hence the microbial loop, in the Bohai and Yellow Seas. In addition, this study also indicates that it is important to take seasonality into account in addressing spatial distribution patterns of marine protists, as previously demonstrated for freshwater protists [64].

## Supporting Information

Figure S1 **Rarefaction curves of operational taxonomic units (OTUs) derived from the three clone libraries of the summertime samples collected from stations H8, B4 and B38. OTUs were defined at a cutoff of 98% sequence identity.** Bars show the 95% confidence intervals.
(TIFF)

Table S1 **Description of samples collected from the Bohai Sea (BS), North Yellow Sea (NYS), and South Yellow Sea (SYS) basins during the summer and winter of 2011.**
(DOC)

Table S2 **List of closest cultured matches of BLAST against GenBank using representative sequences of nanociliate OTUs.**
(DOC)
