# Supplementary figures and images for: SSU rDNA Sequence Diversity and Seasonally Differentiated Distribution of Nanoplanktonic Ciliates in Neritic Bohai and Yellow Seas as Revealed by T-RFLP
Source: PLoS One. 2014 Jul 15;9(7):e102640. doi: 10.1371/journal.pone.0102640 (PMC4099327; doi:10.1371/journal.pone.0102640)

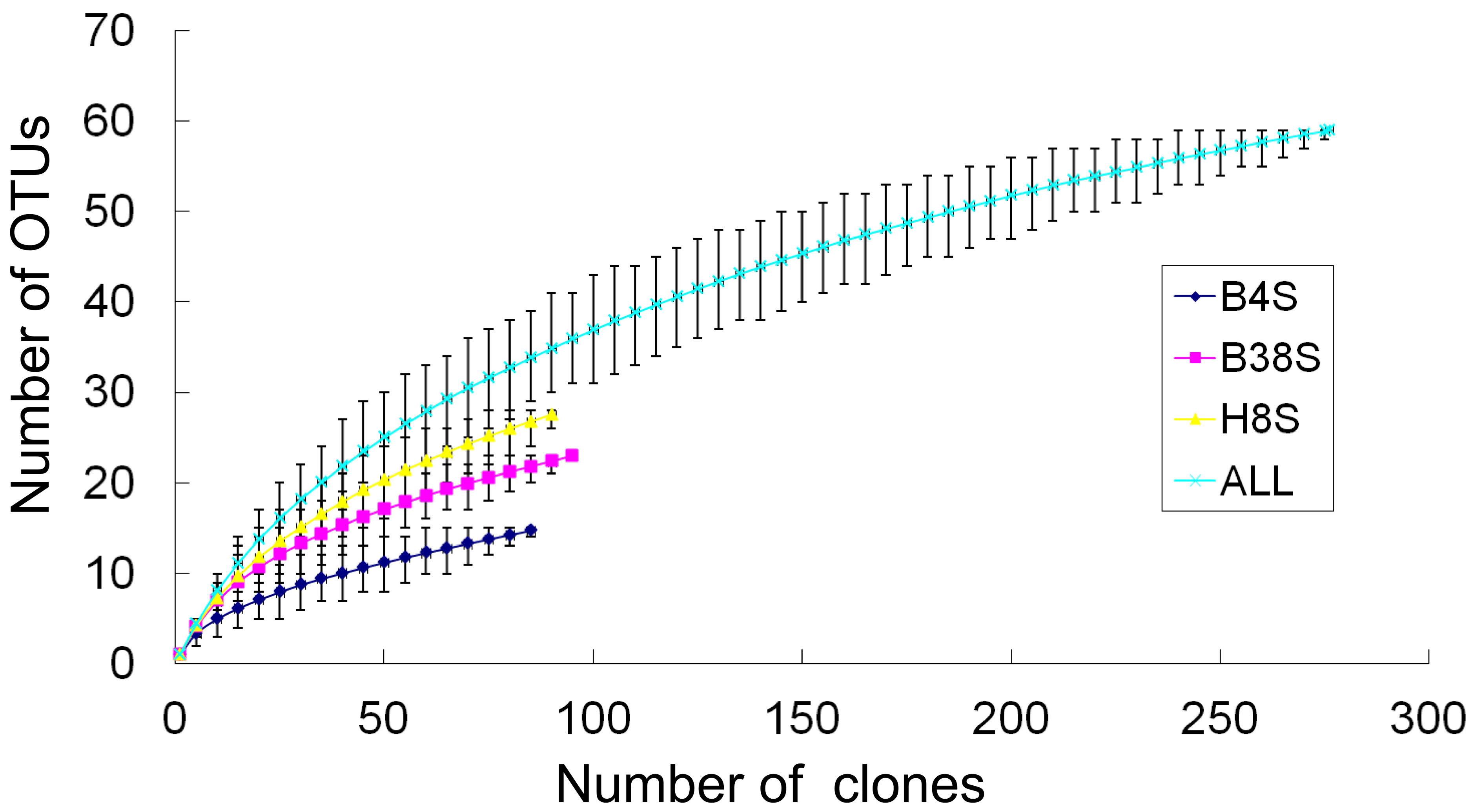

Supplement: Figure S1 — Rarefaction curves of operational taxonomic units (OTUs) derived from the three clone libraries of the summertime samples collected from stations H8, B4 and B38. OTUs were defined at a cutoff of 98% sequence identity. Bars show the 95% confidence intervals. (TIFF) [file pone.0102640.s001.tif]
