# Supplementary material for: SSU rDNA Sequence Diversity and Seasonally Differentiated Distribution of Nanoplanktonic Ciliates in Neritic Bohai and Yellow Seas as Revealed by T-RFLP
Source: PLoS One. 2014 Jul 15;9(7):e102640. doi: 10.1371/journal.pone.0102640 (PMC4099327; doi:10.1371/journal.pone.0102640)
Supplement: Table S1 — Description of samples collected from the Bohai Sea (BS), North Yellow Sea (NYS), and South Yellow Sea (SYS) basins during the summer and winter of 2011. (DOC) [file pone.0102640.s002.doc]

**Table S1.** Description of samples collected from the Bohai Sea (BS), North Yellow Sea (NYS), and South Yellow Sea (SYS) basins during the summer and winter of 2011.

| Basin | Station ID | Latitude  (N) | Longitude (E) | Sample  ID | Date | Time  (hours) | Max water  depth (m) | Temp  (℃) | Salinity  (psu) | pH | DO  (μg/L) | Chl-a  (μg/L) | NO_3_-N  (μmol/L) | NO_2_-N  (μmol/L) | NH_4_-N  (μmol/L) | PO_4_-P  (μmol/L) | SiO_2_  (μmol/L) | N:P | P:Si |
| --- | --- | --- | --- | --- | --- | --- | --- | --- | --- | --- | --- | --- | --- | --- | --- | --- | --- | --- | --- |
| **Summer** |  |  |  |  |  |  |  |  |  |  |  |  |  |  |  |  |  |  |  |
| SYS | H8 | 123°29.730’ | 35°57.780’ | H8S | 14-Jun | 0932 | 75 | 17.09 | 32.35 | 7.98 | 222.47 | 1.12 | 1.02 | 0.18 | 10.25 | 0.01 | 0.23 | 1203.03 | 0.04 |
|  | H9 | 123°30.154’ | 35°30.168’ | H9S | 14-Jun | 1241 | 76 | 18.31 | 32.20 | 7.98 | 215.40 | 0.84 | 1.54 | 0.21 | 12.54 | 0.03 | 0.28 | 477.66 | 0.11 |
|  | H11 | 123°00.059’ | 34°59.765’ | H11S | 14-Jun | 1843 | 72 | 19.10 | 31.97 | 7.98 | 220.06 | 1.50 | 1.10 | 0.29 | 6.71 | 0.04 | 0.48 | 209.13 | 0.08 |
|  | B4 | 122°35.546’ | 36°49.778’ | B4S | 21-Jun | 1003 | 37 | 15.05 | 31.55 | 7.91 | 243.95 | 3.57 | 1.06 | 0.24 | 9.51 | 0.03 | 0.35 | 328.55 | 0.09 |
|  | B10 | 123°59.552’ | 36°59.060’ | B10S | 21-Jun | 1948 | 77 | 21.03 | 31.95 | 7.99 | 204.13 | 0.83 | 1.09 | 0.20 | 8.86 | 0.04 | 0.47 | 226.30 | 0.10 |
| NYS | B17 | 124°05.180’ | 39°12.504’ | B17S | 22-Jun | 1303 | 41 | 11.30 | 31.62 | 7.89 | 260.47 | 4.11 | 0.99 | 0.21 | 9.59 | 0.09 | 0.18 | 117.91 | 0.50 |
|  | B19 | 123°21.567’ | 38°44.470’ | B19S | 22-Jun | 1853 | 57 | 19.74 | 31.57 | 8.01 | 216.04 | 2.15 | 0.94 | 0.20 | 5.73 | 0.09 | 0.13 | 76.17 | 0.68 |
|  | B22 | 122°29.992’ | 38°44.874’ | B22S | 22-Jun | 2349 | 55 | 15.50 | 31.01 | 7.98 | 256.95 | 3.36 | 1.01 | 0.21 | 9.86 | 0.09 | 0.40 | 118.64 | 0.23 |
|  | B24 | 122°29.021’ | 38°09.409’ | B24S | 23-Jun | 1324 | 52 | 18.66 | 31.22 | 8.02 | 222.15 | 4.50 | 1.54 | 0.40 | 4.39 | 0.13 | 0.57 | 50.37 | 0.22 |
|  | B30 | 121°59.871’ | 38°11.923’ | B30S | 24-Jun | 0148 | 56 | 18.97 | 31.18 | 7.98 | 217.30 | 4.47 | 1.06 | 0.26 | 10.90 | 0.09 | 0.33 | 132.71 | 0.28 |
|  | B31 | 121°59.885’ | 38°29.590’ | B31S | 24-Jun | 0410 | 53 | 19.44 | 31.14 | 7.98 | 209.38 | 2.82 | 1.10 | 0.25 | 7.32 | 0.08 | 0.32 | 105.32 | 0.26 |
|  | B33 | 121°37.511’ | 38°40.005’ | B33S | 24-Jun | 0754 | 61 | 18.06 | 30.82 | 7.98 | 221.37 | 2.39 | 1.00 | 0.21 | 7.20 | 0.08 | 0.35 | 106.41 | 0.23 |
|  | B38 | 121°09.491’ | 37°54.656’ | B38S | 24-Jun | 1532 | 22 | 15.25 | 31.18 | 7.96 | 251.21 | 3.47 | 0.96 | 0.19 | 13.53 | 0.07 | 0.13 | 202.99 | 0.54 |
| BS | B39 | 120°44.448’ | 38°20.887’ | B39S | 24-Jun | 2021 | 29 | 13.82 | 31.19 | 7.92 | 240.66 | 4.50 | 1.25 | 0.23 | 4.71 | 0.08 | 0.97 | 77.46 | 0.08 |
|  | B41 | 120°12.018’ | 38°20.079’ | B41S | 24-Jun | 2351 | 28 | 15.70 | 31.03 | 7.97 | 243.99 | 4.48 | 1.10 | 0.17 | 7.54 | 0.07 | 0.38 | 119.81 | 0.19 |
|  | B43 | 119°26.446’ | 38°19.751’ | B43S | 25-Jun | 0742 | 24 | 18.81 | 30.92 | 7.96 | 226.94 | 4.50 | 2.89 | 0.30 | 8.37 | 0.08 | 0.72 | 150.86 | 0.11 |
|  | B60 | 120°14.321’ | 39°19.164’ | B60S | 26-Jun | 1526 | 25 | 17.92 | 31.21 | 7.99 | 215.49 | 4.50 | 1.14 | 0.26 | 7.08 | 0.09 | 1.23 | 98.93 | 0.07 |
| **Winter** |  |  |  |  |  |  |  |  |  |  |  |  |  |  |  |  |  |  |  |
| SYS | H8 | 123°29.570’ | 35°57.605’ | H8W | 4-Dec | 0810 | 75 | 14.09 | 31.79 | 7.96 | 119.07 | 1.53 | 1.45 | 0.42 | 3.00 | 0.08 | 1.82 | 58.54 | 0.05 |
|  | H9 | 123°29.959’ | 35°30.137’ | H9W | 4-Dec | 1130 | 75 | 14.06 | 32.00 | 7.96 | 118.55 | 1.56 | 1.15 | 0.17 | 0.00 | 0.07 | 2.62 | 17.66 | 0.03 |
|  | H11 | 122°59.947’ | 34°59.888’ | H11W | 4-Dec | 1740 | 72 | 14.73 | 31.78 | 7.96 | 117.37 | 1.24 | 1.47 | 0.41 | 6.26 | 0.07 | 1.78 | 124.94 | 0.04 |
|  | B4 | 122°35.557’ | 36°49.709’ | B4W | 21-Nov | 1209 | 38 | 15.18 | 30.90 | 7.91 | 119.51 | 1.11 | 3.18 | 0.46 | 301.06 | 0.06 | 1.45 | 4904.13 | 0.04 |
|  | B10 | 123°59.816’ | 36°59.346’ | B10W | 21-Nov | 2135 | 77 | 14.23 | 31.57 | 7.94 | 121.98 | 1.48 | 2.26 | 0.33 | 5.69 | 0.07 | 1.97 | 114.44 | 0.04 |
| NYS | B12 | 123°03.726’ | 37°53.693’ | B12W | 22-Nov | 0550 | 61 | 13.67 | 30.88 | 7.93 | 122.35 | 1.58 | 1.91 | 0.54 | 57.99 | 0.06 | 1.42 | 974.85 | 0.04 |
|  | B19 | 123°22.118’ | 38°44.051’ | B19W | 22-Nov | 2230 | 57 | 11.48 | 31.69 | 7.79 | 122.75 | 2.72 | 3.22 | 0.41 | 1.48 | 0.08 | 3.70 | 62.73 | 0.02 |
|  | B24 | 122°29.276’ | 38°09.764’ | B24W | 24-Nov | 1212 | 50 | 12.39 | 31.03 | 7.88 | 125.06 | 2.43 | 2.08 | 0.35 | 5.12 | 0.06 | 1.80 | 122.67 | 0.03 |
|  | B28 | 121°59.563’ | 37°42.049’ | B28W | 24-Nov | 1945 | 24 | 12.13 | 30.97 | 7.84 | 128.39 | 1.33 | 3.27 | 0.44 | 3.41 | 0.07 | 2.93 | 96.37 | 0.03 |
|  | B31 | 121°59.878’ | 38°29.596’ | B31W | 25-Nov | 0153 | 50 | 13.26 | 30.89 | 7.85 | 119.94 | 0.97 | 3.00 | 0.58 | 53.03 | 0.08 | 2.53 | 721.64 | 0.03 |
|  | B33 | 121°37.547’ | 38°40.048’ | B33W | 25-Nov | 0525 | 60 | 12.35 | 30.92 | 7.85 | 121.96 | 1.09 | 3.04 | 0.59 | 2.59 | 0.10 | 3.50 | 59.57 | 0.03 |
|  | B36 | 121°16.020’ | 38°15.958’ | B36W | 25-Nov | 0926 | 42 | 12.99 | 30.97 | 7.83 | 119.25 | 0.80 | 3.78 | 0.62 | 2.65 | 0.10 | 4.17 | 67.62 | 0.03 |
|  | B38 | 121°09.594’ | 37°54.632’ | B38W | 25-Nov | 1242 | 22 | 12.50 | 30.93 | 7.85 | 128.76 | 0.96 | 2.81 | 0.34 | 11.47 | 0.09 | 2.25 | 164.86 | 0.04 |
| BS | B39 | 120°44.426’ | 38°20.699’ | B39W | 25-Nov | 1750 | 29 | 13.33 | 31.01 | 7.88 | 124.18 | 0.69 | 4.98 | 0.71 | 36.58 | 0.10 | 2.03 | 442.11 | 0.05 |
|  | B41 | 120°11.523’ | 38°20.133’ | B41W | 25-Nov | 2113 | 28 | 12.71 | 31.20 | 7.95 | 127.41 | 0.92 | 1.90 | 0.72 | 6.32 | 0.07 | 0.38 | 122.48 | 0.19 |
|  | B43 | 119°27.100’ | 38°20.197’ | B43W | 26-Nov | 0532 | 25 | 12.47 | 30.96 | 7.90 | 125.23 | 0.52 | 2.93 | 1.50 | 6.12 | 0.09 | 1.78 | 122.69 | 0.05 |
|  | B47 | 118°58.429’ | 38°39.889’ | B47W | 26-Nov | 1146 | 26 | 12.93 | 30.87 | 7.91 | 125.15 | 0.54 | 4.75 | 0.32 | 19.05 | 0.08 | 1.42 | 284.95 | 0.06 |
|  | B60 | 120°14.362’ | 39°19.143’ | B60W | 27-Nov | 0614 | 25 | 11.27 | 31.25 | 7.88 | 130.25 | 0.46 | 2.31 | 0.66 | 21.79 | 0.08 | 1.00 | 307.84 | 0.08 |
