# Supplementary material for: SSU rDNA Sequence Diversity and Seasonally Differentiated Distribution of Nanoplanktonic Ciliates in Neritic Bohai and Yellow Seas as Revealed by T-RFLP
Source: PLoS One. 2014 Jul 15;9(7):e102640. doi: 10.1371/journal.pone.0102640 (PMC4099327; doi:10.1371/journal.pone.0102640)
Supplement: Table S2 — List of closest cultured matches of BLAST against GenBank using representative sequences of nanociliate OTUs. (DOC) [file pone.0102640.s003.doc]

**Table S2.** List of closest cultured matches of BLAST against GenBank using representative sequences of nanociliate OTUs.

| OTU ID | Number of sequence | Representative  clone | Closet cultured match (accession No.) | Query  cover | Identity | Taxonomy |
| --- | --- | --- | --- | --- | --- | --- |
|  |  |  |  |  |  | OLIGOTRICHIA |
| OTU1 | 38 | B4S-6 | *Strombidium* cf. *basimorphum* (JF791016) | 100% | 99.6% | Strombidiidae |
| OTU8 | 11 | B38S-45 | *Strombidium* cf. *basimorphum* (JF791016) | 100% | 98.0% | Strombidiidae |
| OTU9 | 9 | B38S-15 | *Strombidium* cf. *basimorphum* (JF791016) | 100% | 98.2% | Strombidiidae |
| OTU13 | 5 | B4S-32 | *Strombidium* cf. *basimorphum* (JF791016) | 100% | 97.8% | Strombidiidae |
| OTU23 | 3 | H8S-32 | *Strombidium* cf. *basimorphum* (JF791016) | 100% | 96.7% | Strombidiidae |
| OTU27 | 2 | B4S-44 | *Strombidium* cf. *basimorphum* (JF791016) | 100% | 98.4% | Strombidiidae |
| OTU29 | 2 | B4S-66 | *Strombidium* cf. *basimorphum* (JF791016) | 100% | 98.0% | Strombidiidae |
| OTU24 | 2 | B4S-14 | *Strombidium* cf. *basimorphum* (JF791016) | 100% | 97.2% | Strombidiidae |
| OTU33 | 2 | H8S-33 | *Strombidium* cf. *basimorphum* (JF791016) | 100% | 97.0% | Strombidiidae |
| OTU38 | 1 | B4S-24 | *Strombidium* cf. *basimorphum* (FJ480419) | 100% | 96.1% | Strombidiidae |
| OTU52 | 1 | H8S-26 | *Strombidium* cf. *basimorphum* (JF791016) | 100% | 97.1% | Strombidiidae |
| OTU43 | 1 | B38S-37 | *Strombidium* cf. *basimorphum* (JF791016) | 100% | 96.5% | Strombidiidae |
| OTU44 | 1 | B38S-42 | *Strombidium* cf. *basimorphum* (JF791016) | 100% | 95.5% | Strombidiidae |
| OTU10 | 8 | B4S-18 | *Strombidium biarmatum* (AY541684) | 100% | 97.6% | Strombidiidae |
| OTU3 | 16 | B38S-1 | *Spirostrombidium* sp. (JN712658) | 100% | 97.2% | Strombidiidae |
| OTU19 | 3 | B38S-21 | *Spirostrombidium* sp. (JN712658) | 100% | 98.6% | Strombidiidae |
| OTU21 | 3 | B38S-71 | *Spirostrombidium* sp. (JN712658) | 100% | 96.5% | Strombidiidae |
| OTU25 | 2 | B4S-19 | *Spirostrombidium* sp. (JN712658) | 100% | 94.5% | Strombidiidae |
| OTU46 | 1 | B38S-104 | *Spirostrombidium* sp. (JN712658) | 100% | 96.3% | Strombidiidae |
| OTU59 | 1 | H8S-143 | *Spirostrombidium* sp. (JN712658) | 100% | 95.9% | Strombidiidae |
| OTU47 | 1 | B38S-108 | *Spirostrombidium* sp. (JN712658) | 99% | 94.1% | Strombidiidae |
| OTU4 | 15 | B38S-67 | *Strombidium* sp. (AY143564) | 100% | 98.6% | Strombidiidae |
| OTU7 | 13 | B38S-4 | *Strombidium* sp. (AY143564) | 100% | 98.2% | Strombidiidae |
| OTU15 | 4 | B38S-22 | *Strombidium* sp. (AY143564) | 100% | 97.4% | Strombidiidae |
| OTU26 | 2 | B4S-35 | *Strombidium* sp. (AY143564) | 100% | 98.6% | Strombidiidae |
| OTU37 | 2 | H8S-108 | *Strombidium* sp. (AY143564) | 100% | 97.0% | Strombidiidae |
| OTU42 | 1 | B38S-28 | *Strombidium* sp. (AY143564) | 100% | 97.6% | Strombidiidae |
| OTU51 | 1 | H8S-19 | *Strombidium* sp. (AY143564) | 100% | 97.2% | Strombidiidae |
| OTU58 | 1 | H8S-119 | *Strombidium* sp. (AY143564) | 100% | 96.7% | Strombidiidae |
| OTU31 | 2 | B38S-8 | *Varistrombidium kielum* (DQ811090 ) | 100% | 98.4% | Strombidiidae |
| OTU41 | 1 | B38S-26 | *Varistrombidium kielum* (DQ811090 ) | 100% | 96.3% | Strombidiidae |
| OTU5 | 14 | B4S-43 | *Laboea strobila* (AY302563) | 100% | 97.8% | Strombidiidae |
| OTU14 | 5 | B38S-14 | *Laboea strobila* (AY302563) | 100% | 97.7% | Strombidiidae |
| OTU20 | 3 | B38S-38 | *Laboea strobila* (AY302563) | 100% | 96.5% | Strombidiidae |
| OTU16 | 4 | H8S-5 | *Laboea strobila* (AY302563) | 100% | 95.3% | Strombidiidae |
| OTU56 | 1 | H8S-110 | *Strombidium* sp. (AY143565) | 100% | 94.7% | Strombidiidae |
| OTU17 | 3 | B38S-11 | *Strombidium* sp. (AY143565) | 100% | 93.7% | Strombidiidae |
| OTU18 | 3 | B38S-16 | *Novistrombidium sinicum* (FJ422990) | 100% | 96.5% | Strombidiidae |
| OTU6 | 13 | B38S-69 | *Pseudotontonia simplicidens* (FJ422993) | 100% | 98.6% | Tontoniidae |
| OTU34 | 2 | H8S-37 | *Pseudotontonia simplicidens* (FJ422993) | 100% | 98.4% | Tontoniidae |
| OTU36 | 2 | H8S-99 | *Pseudotontonia simplicidens* (FJ422993) | 100% | 96.7% | Tontoniidae |
| OTU50 | 1 | H8S-12 | *Pseudotontonia simplicidens* (FJ422993) | 100% | 96.7% | Tontoniidae |
| OTU32 | 2 | B38S-60 | *Pseudotontonia simplicidens* (FJ422993) | 100% | 96.5% | Tontoniidae |
| OTU55 | 1 | H8S-90 | *Pseudotontonia simplicidens* (FJ422993) | 100% | 95.7% | Tontoniidae |
| OTU53 | 1 | H8S-28 | *Pseudotontonia simplicidens* (FJ422993) | 100% | 95.5% | Tontoniidae |
|  |  |  |  |  |  | CHOREOTRICHIA |
| OTU2 | 35 | B4S-2 | *Rimostrombidium veniliae* (FJ876964) | 100% | 96.6% | Strobilidiidae |
| OTU40 | 1 | B4S-128 | *Rimostrombidium veniliae* (FJ876964) | 100% | 96.7% | Strobilidiidae |
| OTU28 | 2 | B4S-57 | *Pelagostrobilidium paraepacrum* (FJ876963) | 100% | 96.7% | Strobilidiidae |
| OTU11 | 8 | H8S-6 | *Lynnella semiglobulosa* (FJ876965) | 100% | 98.8% | Lynnellidae |
| OTU22 | 3 | H8S-27 | *Lynnella semiglobulosa* (FJ876965) | 100% | 96.3% | Lynnellidae |
| OTU35 | 2 | H8S-47 | *Lynnella semiglobulosa* (FJ876965) | 100% | 96.1% | Lynnellidae |
| OTU30 | 2 | B4S-71 | *Tintinnopsis karajacensis* (AB640663) | 100% | 98.6% | Codonellidae |
| OTU39 | 1 | B4S-28 | *Codonaria cistellula* (JQ408202) | 100% | 97.8% | Codonellidae |
| OTU54 | 1 | H8S-51 | *Codonellopsis gaussi* (JQ924053) | 100% | 96.0% | Codonellopsidae |
| OTU12 | 7 | B4S-5 | *Amphorellopsis quinquealata* (JQ924058) | 100% | 96.0% | Tintinnidae |
| OTU45 | 1 | B38S-81 | *Amphorellopsis quinquealata* (JQ924058) | 100% | 96.4% | Tintinnidae |
| OTU48 | 1 | B38S-110 | *Amphorellopsis quinquealata* (JQ924058) | 100% | 94.7% | Tintinnidae |
| OTU49 | 1 | H8S-3 | *Salpingella acuminate* (Q408155) | 100% | 99.2% | Tintinnidae |
| OTU57 | 1 | H8S-111 | *Salpingella acuminate* (Q408155) | 100% | 96.4% | Tintinnidae |
